# Alcohol relapse and its predictors after liver transplantation for alcoholic liver disease: a systematic review and meta-analysis

**DOI:** 10.1186/s12876-019-1050-9

**Published:** 2019-08-22

**Authors:** Lancharat Chuncharunee, Noriyo Yamashiki, Ammarin Thakkinstian, Abhasnee Sobhonslidsuk

**Affiliations:** 1 Division of Gastroenterology and Hepatology, Department of Medicine, Faculty of Medicine Ramathibodi Hospital, Mahidol University, 270 Rama 6 Road, Bangkok, 10400 Thailand; 2 Organ Transplantation Unit, Kyoto University, Kyoto, Japan; 3 Section for Clinical Epidemiology and Biostatistics, Faculty of Medicine Ramathibodi Hospital, Mahidol University, Bangkok, Thailand

**Keywords:** Liver transplantation, Alcohol, Relapse, Recidivism, Psychiatric comorbidity

## Abstract

**Background:**

Alcoholic liver disease (ALD) is the leading cause of liver transplantation (LT). The magnitude and risk factors of post-LT alcohol relapse are not well described. We conducted a meta-analysis to evaluate alcohol relapse rate and its predictors after LT.

**Methods:**

Searches of MEDLINE and SCOPUS identified eligible published studies of alcohol relapse after LT published up to 31 March 2018. Alcohol relapse was defined as any alcohol consumption post-LT, and heavy alcohol relapse was defined as a relapse of alcohol consumption that was associated with a significant harm. Data for the proportion of alcohol relapse was pooled using a meta-analysis for pooling proportion. An odds ratio (OR) of the predictor of alcohol relapse was extracted and pooled using meta-analysis for the pooling risk factor. Data were analyzed using a random effect model if heterogeneity was presented; otherwise, a fixed effect model was applied. The study was registered at PROSPERO (CRD42017052659).

**Results:**

Ninety-two studies with over 8000 cases were recruited for pooling proportion of alcohol relapse. The alcohol relapse rate and heavy alcohol relapse rate after LT during the mean follow-up time of 48.4 ± 24.7 months were 22% (95% confidence interval (CI): 19–25%) and 14% (95%CI: 12–16%). Psychiatric comorbidities (odds ratio (OR) 3.46, 95%CI: 1.87–6.39), pre-transplant abstinence of less than 6 months (OR 2.76, 95%CI: 2.10–3.61), unmarried status (OR 1.84, 95%CI: 1.39–2.43), and smoking (OR 1.72, 95%CI: 1.21–2.46) were associated with alcohol relapse after LT. However, we noticed publication bias of unpublished negative studies and high heterogeneity of results.

**Conclusions:**

Post-transplant alcohol relapse occurred in about one-fifth of patients who underwent alcohol-related LT. Psychiatric comorbidities represented the strongest predictor of alcohol relapse. Psychiatric comorbidities monitoring and pre-LT alcohol abstinence for at least 6 months may decrease alcohol relapse after LT.

## Background

Chronic and excessive alcohol consumption is a major cause of death around the world. Regular alcohol consumption can lead to steatosis, steatohepatitis, liver cirrhosis, and hepatocellular carcinoma [1–3]. Liver transplantation (LT) is an extended treatment for end-stage liver diseases; alcoholic liver cirrhosis is the second most frequent cause for LT in the United States and in Europe [4]. Previous studies demonstrated that LT in ALD patients offers an equal survival rate as that in other causes of end-stage liver disease [5]. Furthermore, LT for severe alcoholic hepatitis has a favorable outcome and better survival than medical therapy, but non-surgical therapy remains the standard of care for patients with severe alcoholic hepatitis [6, 7].

The issues of recidivism and disease recurrence remain a concern in LT for alcoholic liver disease. Alcohol relapse negatively impacts outcomes including graft rejection and graft loss from poor medical compliance, post-transplant malignancy, cardiovascular diseases, alcoholic cirrhosis, and decreased long-term survival [8–11]. An abstinence period of at least 6 months before LT is a mandatory selection criterion in most liver transplant centers, but the benefit of such pre-transplant 6 month abstinence remains unclear [8, 12, 13]. Furthermore, there are subsequent reports indicating that an abstinence period of 6 months is not a significant predictive factor for recidivism [14–16]. Careful evaluation of patients with alcoholic liver disease prior to liver transplantation can identify patients with a high risk of alcohol relapse. Modifying the negative factors before LT can prevent alcohol relapse and improve post-transplant survival.

Most of these studies on alcohol recidivism after LT were done in single centers and were reported as descriptive data [16–24]. A previously published meta-analysis study of alcohol relapse after liver transplantation by Dew et al. in 2008 only included published reports on this topic up to 2004 [25]. Several predictive factors have been reported in the last decade [8, 14, 15, 25, 26]. Thus, we performed a systematic review and meta-analysis from the published literature with the following objectives: First, to pool prevalence of alcohol relapse after LT; second, to explore factors associated with alcohol relapse and pool their magnitude of effects in alcoholic liver disease patients with LT.

## Methods

This meta-analysis was conducted by following the Preferred Reporting Items for Systematic Reviews and Meta-analysis (PRISMA) guidelines, and the review protocol was registered at PROSPERO (CRD42017052659).

### Search strategy

Two investigators (L.C. and A.S.) independently conducted a search of databases via MEDLINE and SCOPUS via PubMed and Scopus search engines to identify relevant studies published up to 31 March 2018. The search terms were constructed by domains of patients, intervention/exposure, and outcome. The search strategy is outlined in Table 1. The investigators supplemented the manual reviews of article reference lists to identify studies that had not been included from the initial database search and also performed manual reviews of the relevant studies.

**Table 1:** Search terms and search strategy

Domain	Search term	Search strategy
P-Patient	- “Alcoholic hepatitis”	#1
	- “Alcoholic liver disease”	#2
	- “Alcoholic cirrhosis”	#3
	- “Liver transplantation”	#4
	- “Hepatic transplantation”	#5
All P	#6	#1 OR #2 OR #3 AND #4 OR #5
E-Exposure (I-intervention/C-comparator)	- Gender	#7
	- Sex	#8
	#9	#7 OR #8
	- Age	#10
	- “Marital status”	#11
	- Divorced	#12
	#13	#11 OR #12
	- “Socioeconomic status”	#14
	- Income	#15
	- Education	#16
	#17	#14 OR #15 OR #16
	- “Alcohol dependence”	#18
	- Depression	#19
	- “Drug use”	#20
	- “Substance use”	#21
	- “Substance abuse”	#22
	- substance	#23
	#24	#20 OR #21 OR #22 OR #23
	- Family history of alcohol	#25
	- Alcohol abstinence	#26
	- Alcohol cessation	#27
	- Alcohol quit	#28
	- Alcohol stop	#29
	- Alcohol sobriety	#30
	#31	#26 OR #27 OR #28 OR #29 OR #30
	- Rehabilitation	#32
	- High Risk Alcoholism Relapse	#33
	- HRAR	#34
	#35	#33 OR #34
All E	#36	#9 OR #10 OR #13 OR #17 OR #18 OR #19 OR #24 OR #25 OR #31 OR #32 OR #35
O-Outcome	- Alcohol relapse	#37
	- Alcohol recurrence	#38
	- Recidivism	#39
All O	#40	#37 OR #38 OR #39
Overall		#6 AND #36 AND #40

### Study selection

Two investigators (L.C. and A.S.) determined the eligibility of each article for inclusion by screening for relevance on titles and abstracts in parallel. If an eligibility of the study was indeterminable from abstracts, then the full articles were retrieved. The kappa statistic was used to estimate the agreement between the two reviewers [27]. Discordant decisions between the two investigators were advanced to full-text review and resolved by consensus with the third investigator (A.T.).

### Inclusion criteria

#### Studies were eligible if they met all of the following criteria

- The study was an observational design including cohort, cross-sectional, or case-control study published as an original article.
- The work studied adult patients aged over 18 years with alcoholic liver disease who underwent any type of LT.
- Reporting any of the following outcomes: alcohol relapse or alcohol recurrence.
- The study assessed association between alcohol relapse and any risk factor including seven domains as follows: demographic data, psychiatric conditions, socioeconomic status, family support, alcohol abstinence, rehabilitation program, and high-risk alcohol relapse (HRAR) scale [28].

#### Exclusion criteria

Studies were excluded if they met one of the below criteria:

- Non-English articles that cannot be translated.
- Studies with multi-organ transplantation.
- Insufficient data for extraction.

#### Outcome of interest

The outcome of interest was alcohol relapse and heavy alcohol relapse in patients who underwent LT for alcoholic liver disease. We selected papers on the occurrence of alcohol relapse based on the original authors’ definition of alcohol relapse and heavy relapse and used several methods of relapse assessment such as self-reporting and collateral reporting except for biochemical testing and indirect measures. In general, alcohol relapse was defined as any alcohol consumption post-transplantation, and heavy alcohol relapse was defined as a relapse of alcohol consumption associated with significant medical or social harm [29].

#### Data extraction

Data obtained from each study was independently extracted by two reviewers (L.C. and A.S.) using a standardized extraction form. Study design, details of the publication, the number of subjects, and baseline characteristics of study populations were extracted including patients with alcohol relapse and predictive factors of alcohol relapse after LT.

#### Quality and risk of bias assessment

All selected studies were independently reviewed by two investigators (L.C. and A.S.). Disagreements between the two reviewers were resolved by consensus with the third investigator (A.T.). Quality was assessed using a Newcastle-Ottawa Scale for eligible studies including selection, exposure, and comparability of studies on the basis of the design or analysis and assessment of the outcomes (Table 2).

**Table 2:** Newcastle-Ottawa quality assessment scale of each included studies

Author	Year	S1	S2	S3	S4	C	O1	O2	O3
Starzl TE [30]	1988	✹	✹		✹	✹			✹
Bird GLA [12]	1990	✹	✹	✹	✹	✹	✹		✹
Kumar S [31]	1990	✹	✹	✹	✹	✹✹	✹		✹
Doffoel M[32]	1992	✹	✹		✹	✹			✹
Knechtle SJ [33]	1992	✹	✹	✹	✹	✹	✹	✹	
Lucey MR [34]	1992	✹	✹	✹	✹	✹✹	✹	✹	✹
Berlakovich GA [35]	1994	✹	✹	✹	✹	✹	✹		✹
Howard L [36]	1994	✹	✹	✹	✹	✹	✹		✹
Osorio RW [37]	1994	✹	✹	✹	✹	✹✹	✹		✹
Gerhardt TC [38]	1996	✹	✹	✹	✹	✹✹	✹		✹
Tringali RA [39]	1996	✹	✹	✹	✹	✹✹	✹		✹
Tripp LE [40]	1996	✹	✹		✹	✹			✹
Zibari GB [41]	1996	✹	✹	✹	✹	✹	✹		✹
Anand AC [42]	1997	✹	✹	✹	✹	✹	✹		✹
Coffman KL [43]	1997	✹	✹		✹	✹			✹
Everson G [44]	1997	✹	✹	✹	✹	✹	✹		
Foster PF [24]	1997	✹	✹	✹	✹	✹	✹	✹	✹
Lucey MR [45]	1997	✹	✹	✹	✹	✹	✹	✹	✹
Reeck UH [46]	1997	✹	✹		✹	✹			
Shakil AO [47]	1997	✹	✹		✹	✹			✹
Stefanini GF [48]	1997	✹	✹		✹	✹			✹
DiMartini A [49]	1998	✹	✹		✹	✹			
Fabrega E [50]	1998	✹	✹	✹	✹	✹	✹		✹
Heinemann A [51]	1998	✹	✹	✹	✹	✹	✹		✹
Tang H [52]	1998	✹	✹		✹	✹✹			✹
Conjeevaram HS [53]	1999	✹	✹	✹	✹	✹✹	✹		✹
Gledhill J [54]	1999	✹	✹	✹	✹	✹	✹		✹
Newton SE [55]	1999	✹	✹		✹	✹			
Pageaux GP [18]	1999	✹	✹	✹	✹	✹✹	✹		✹
Romano DR [56]	1999	✹	✹	✹	✹	✹	✹	✹	
Abosh D [57]	2000	✹	✹		✹	✹			
Berlakovich GA [58]	2000	✹	✹	✹	✹	✹	✹	✹	✹
Burra P [59]	2000	✹	✹	✹	✹	✹✹	✹		✹
DiMartini A [60]	2000	✹	✹	✹	✹	✹	✹		✹
Jain A [61]	2000	✹	✹	✹	✹	✹✹	✹	✹	✹
Pereira SP [62]	2000	✹	✹	✹	✹	✹	✹		✹
Platz KP [21]	2000	✹	✹	✹	✹	✹	✹		✹
Bellamy CO [63]	2001	✹	✹	✹	✹	✹	✹		✹
DiMartini A [64]	2001	✹	✹	✹	✹	✹	✹		✹
Gish RG [65]	2001	✹	✹	✹	✹	✹✹	✹	✹	✹
Karman JF [66]	2001	✹	✹		✹	✹			✹
Mackie J [15]	2001	✹	✹	✹	✹	✹✹	✹		✹
Tome S [67]	2002	✹	✹		✹	✹			✹
Berlakovich GA [68]	2004	✹	✹	✹	✹	✹	✹		✹
Jauhar S [14]	2004	✹	✹		✹	✹✹			✹
Miguet M [69]	2004	✹	✹	✹	✹	✹	✹		✹
Björnsson E [20]	2005	✹	✹	✹	✹	✹✹	✹		✹
Cuadrado A [10]	2005	✹	✹	✹	✹	✹✹	✹	✹	✹
DiMartini A [70]	2006	✹	✹	✹	✹	✹	✹		✹
Hwang S [71]	2006	✹	✹	✹	✹	✹	✹		✹
Kelly M [72]	2006	✹	✹	✹	✹	✹✹	✹	✹	✹
De Gottardi A [29]	2007	✹	✹	✹	✹	✹✹	✹	✹	✹
Dumortier J [73]	2007	✹	✹	✹	✹	✹	✹	✹	✹
Newton SE [74]	2007	✹	✹		✹	✹		✹	
Nickels M [75]	2007	✹	✹		✹	✹			✹
Pfitzmann R [8]	2007	✹	✹	✹	✹	✹✹	✹	✹	✹
Vieira A [76]	2007	✹	✹		✹	✹			✹
Wells JT [77]	2007	✹	✹	✹	✹	✹	✹	✹	✹
Gedaly R [23]	2008	✹	✹		✹	✹			✹
Immordino G [17]	2009	✹	✹		✹	✹			✹
Tandon P [26]	2009	✹	✹	✹	✹	✹	✹	✹	✹
Biselli M [78]	2010	✹	✹	✹	✹	✹	✹	✹	✹
Chen GH [79]	2010	✹	✹	✹	✹	✹	✹		✹
DiMartini A [80]	2010	✹	✹	✹	✹	✹	✹		✹
Karim Z [81]	2010	✹	✹	✹	✹	✹✹	✹		✹
Hartl J [82]	2011	✹	✹	✹	✹	✹✹	✹		✹
Mathurin P [7]	2011	✹	✹	✹	✹	✹	✹		✹
Schmeding M [9]	2011	✹	✹	✹	✹	✹	✹		✹
Staufer K [83]	2011	✹	✹	✹	✹	✹	✹		✹
Faure S [84]	2012	✹	✹	✹	✹	✹	✹	✹	✹
Addolorato G [85]	2013	✹	✹	✹	✹	✹✹	✹		✹
Deruytter E [86]	2013	✹	✹	✹	✹	✹✹	✹	✹	✹
Kawaguchi Y [87]	2013	✹	✹	✹	✹	✹✹	✹		✹
Park YH [19]	2013	✹	✹		✹	✹✹		✹	✹
Rice JP [88]	2013	✹	✹		✹	✹✹		✹	✹
Rodrigue JR [89]	2013	✹	✹	✹	✹	✹✹	✹	✹	✹
Egawa H [16]	2014	✹	✹	✹	✹	✹✹	✹		✹
Grąt M [90]	2014	✹	✹		✹	✹		✹	✹
Piano S [91]	2014	✹	✹	✹	✹	✹	✹		✹
Dumortier J [92]	2015	✹	✹	✹	✹	✹	✹	✹	✹
Hasanin M [93]	2015	✹	✹		✹	✹		✹	
Satapathy SK [94]	2015	✹	✹		✹	✹		✹	✹
Zhou M [28]	2015	✹	✹	✹	✹	✹✹	✹		✹
Askgaard G [95]	2016	✹	✹	✹	✹	✹✹	✹		✹
Hajifathalian K [96]	2016	✹	✹		✹	✹			✹
Im GY [97]	2016	✹	✹		✹	✹✹			✹
Kollmann D [98]	2016	✹	✹	✹	✹	✹	✹	✹	✹
Lee BP [99]	2017	✹	✹	✹	✹	✹✹	✹		✹
Onishi Y [100]	2017	✹	✹	✹	✹	✹✹	✹	✹	✹
Wigg AJ [101]	2017	✹	✹	✹	✹	✹✹	✹	✹	✹

#### Statistical analysis

The rate of alcohol relapse after LT was estimated along with its 95% confidence interval (CI) for each study. The rate was then pooled across studies using a meta-analysis for pooling proportion [102]. The random effect model was applied if there was heterogeneity between studies; otherwise, a fixed-effect model was applied. An odds ratio (OR) along with 95% CI of risk factor associated with alcohol relapse after LT was estimated for each study. Heterogeneity was assessed using the Cochrane Q test and the I^2^ statistic. Heterogeneity was present when the Q test was significant (*p* < 0.1) or I^2^ ≥ 25%. The sources of heterogeneity were then explored using a meta-regression if the data of the co-variables were available. Subgroup analysis by age, region of study, definition of alcohol relapse, and follow-up time was then performed accordingly. Publication bias was assessed by Egger’s test and a funnel plot. If there was asymmetry suggested from either a funnel plot or Egger’s test, then a contour-enhanced funnel plot was used to explore whether the asymmetry was due to publication bias or heterogeneity. All analyses were performed using STATA software version 14.1. *P*-values < 0.05 and < 0.10 were considered statistically significant for a two-sided test and one-sided test, respectively.

## Results

### Search result

**Fig. 1:**
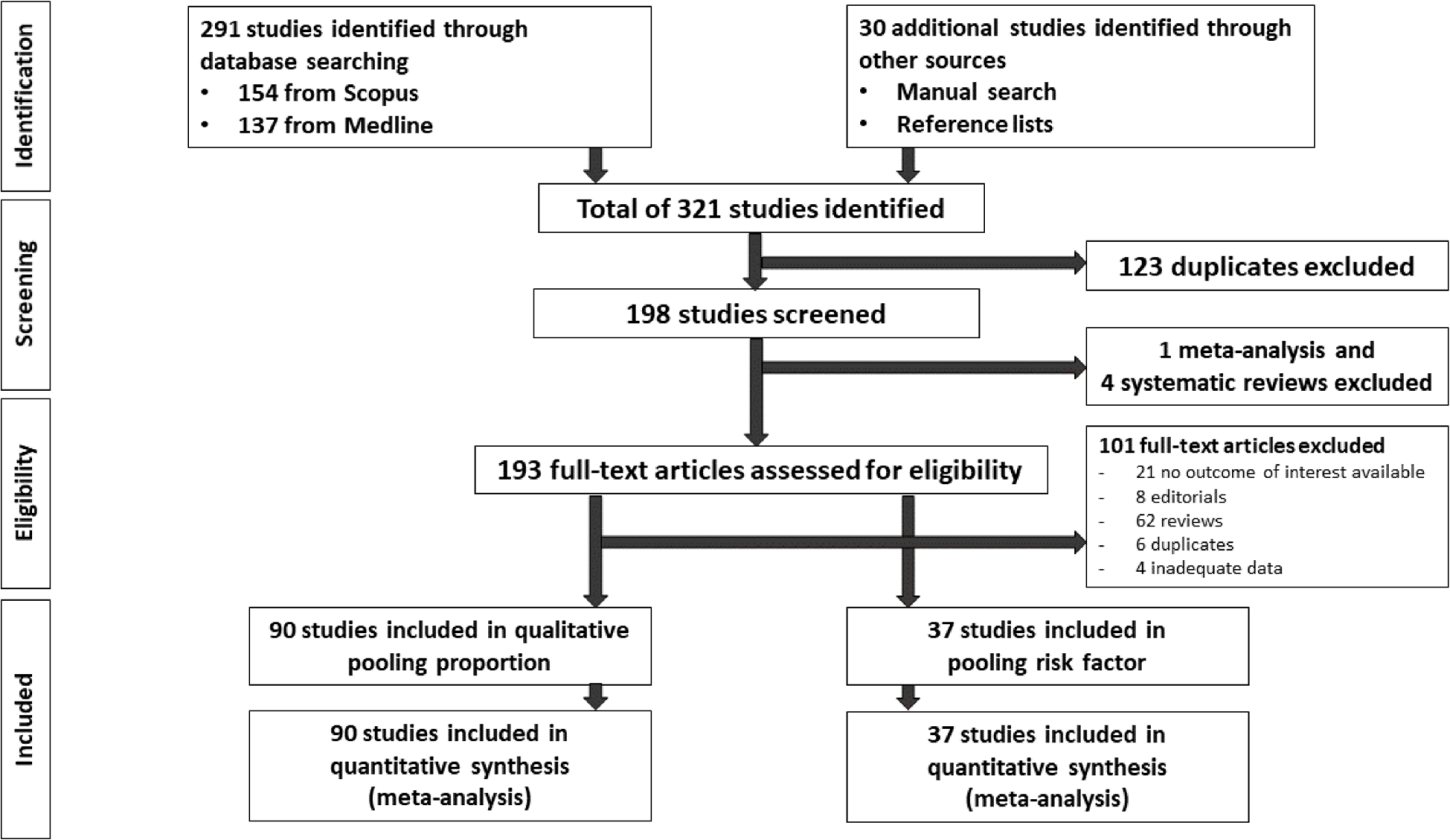
Protocol flow chart

A total of 291 studies were identified from PubMed and Scopus databases plus 30 additional studies from the reference lists (Fig. 1). The title and abstracts were reviewed for 321 studies; 123 duplicated studies, 1 meta-analysis, and 4 systematic reviews were removed [25, 103–106]. The remaining 193 studies were reviewed in full text excluding 101 studies. Of the remaining studies, 90 reported the proportion of alcohol relapse, and 37 studies assessed risk factors of alcohol relapse. The kappa index between the two reviewers (L.C. and A.S.) was 0.96 for data extraction, which indicated very good inter-observer agreement.

### Study characteristics

Of the 90 studies reporting alcohol relapse, 86 were cohort studies (72 retrospective and 14 prospective cohorts), 2 were cross-sectional studies, and 2 were case-control studies (Table 3). Data for the 86 cohort studies were used for pooling in the incidence of alcohol relapse. Of these, 45 cohorts (40 retrospective and 5 prospective cohorts) were pooled for the proportion of heavy alcohol relapse; 37 studies (43.0%) were from North America, 40 studies (46.5%) were from Europe, 8 studies (9.3%) were from Asia-Pacific, and 1 study (0.1%) was from Brazil.

**Table 3:** Main characteristics of the studies included in the meta-analysis

Author	Year	Type of study	Region of study	Mean age (years)	Number of population	Number of any relapse	Number of heavy relapse	Follow-up time (months)
Starzl TE [30]	1988	Retrospective Cohort	US-Canada	-	35	2	-	24
Bird GL [12]	1990	Retrospective Cohort	Europe	-	18	4	-	-
Kumar S [31]	1990	Retrospective Cohort	US-Canada	48.2	52	6	2	25
Doffoel M [32]	1992	Retrospective Cohort	Europe	-	57	19	-	-
Knechtle SJ [33]	1992	Case-control	US-Canada	51	32	4	-	-
Lucey MR [34]	1992	Retrospective Cohort	US-Canada	44	45	5	2	15
Berlakovich GA[35]	1994	Retrospective Cohort	Europe	47.6	44	14	7	33
Howard L [36]	1994	Retrospective Cohort	Europe	50	20	19	16	34
Osorio RW [37]	1994	Prospective Cohort	US-Canada	47	37	7	3	21
Gerhardt TC [38]	1996	Retrospective Cohort	US-Canada	49	41	20	2	47
Tringali RA [39]	1996	Retrospective Cohort	US-Canada	47.4	58	12	10	28
Tripp LE [40]	1996	Retrospective Cohort	US-Canada	49	68	9	5	-
Zibari GB [41]	1996	Retrospective Cohort	US-Canada	47	29	2	-	-
Anand AC [42]	1997	Retrospective Cohort	Europe	47.5	39	5	-	25
Coffman KL [43]	1997	Prospective Cohort	US-Canada	-	91	18	-	-
Everson G [44]	1997	Retrospective Cohort	US-Canada	53	62	11	6	12
Foster PF [24]	1997	Retrospective Cohort	US-Canada	48.6	63	13	-	49.3
Lucey MR [45]	1997	Retrospective Cohort	US-Canada	46	50	17	-	63
Reeck UH [46]	1997	Retrospective Cohort	Europe	-	52	6	-	-
Shakil AO [47]	1997	Retrospective Cohort	US-Canada	41	8	3	-	-
Stefanini GF [48]	1997	Retrospective Cohort	Europe	47	11	3	1	48
DiMartini A [49]	1998	Retrospective Cohort	US-Canada	49.5	63	15	-	-
Fabrega E [50]	1998	Retrospective Cohort	Europe	49	44	8	-	37.8
Heinemann A [51]	1998	Retrospective Cohort	Europe	45.6	13	4	-	-
Tang H [52]	1998	Retrospective Cohort	Europe	48	56	28	9	24
Conjeevaram HS [53]	1999	Retrospective Cohort	US-Canada	47	68	8	8	-
Gledhill J [54]	1999	Retrospective Cohort	Europe	48	31	7	-	13.5
Newton SE [55]	1999	Retrospective Cohort	US-Canada	47	122	33	-	62
Pageaux GP [18]	1999	Retrospective Cohort	Europe	48.8	47	15	5	42.1
Romano DR [56]	1999	Case-control	Europe	47.6	152	7	-	-
Abosh D [57]	2000	Retrospective Cohort	US-Canada	50	10	5	5	10
Berlakovich GA [58]	2000	Retrospective Cohort	Europe	-	118	15	-	53.7
Burra P [59]	2000	Prospective Cohort	Asia Pacific	48	34	11	4	40.1
DiMartini A [60]	2000	Retrospective Cohort	Europe	50	72	4	1	-
Jain A [61]	2000	Retrospective Cohort	US-Canada	50.8	185	37	1	94
Pereira SP [62]	2000	Retrospective Cohort	Europe	51	56	28	15	30
Platz KP [21]	2000	Retrospective Cohort	Europe	-	117	30	-	-
Bellamy CO [63]	2001	Retrospective Cohort	US-Canada	53	123	13	-	84
DiMartini A [64]	2001	Prospective Cohort	US-Canada	-	36	8	-	-
Gish RG [65]	2001	Prospective Cohort	US-Canada	47	61	12	-	82.8
Karman JF [66]	2001	Retrospective Cohort	US-Canada	49	19	4	-	36
Mackie J [15]	2001	Retrospective Cohort	Europe	51	46	21	3	25
Tome S [67]	2002	Prospective Cohort	Europe	51	68	7	2	38
Berlakovich GA [68]	2004	Case-control	Europe	51.5	44	3	-	43.5
Jauhar S [14]	2004	Retrospective Cohort	US-Canada	51	11	17	15	44.1
Miguet M [69]	2004	Prospective Cohort	Europe	48.7	51	13	9	35.7
Björnsson E [20]	2005	Retrospective Cohort	Europe	53	93	32	7	31
Cuadrado A [10]	2005	Retrospective Cohort	Europe	48.9	54	14	14	99.2
DiMartini A [70]	2006	Prospective Cohort	US-Canada	49.7	167	70	43	-
Hwang S [71]	2006	Retrospective Cohort	US-Canada	50	15	3	-	41
Kelly M [72]	2006	Retrospective Cohort	Asia Pacific	50.1	90	28	18	67
De Gottardi A [29]	2007	Retrospective Cohort	Europe	51.3	387	46	46	61.2
Dumortier J [73]	2007	Retrospective Cohort	Europe	50	305	37	37	63
Newton SE [74]	2007	Cross sectional	US-Canada	-	18	4	-	-
Nickels M [75]	2007	Retrospective Cohort	US-Canada	48.8	27	8	-	23.4
Pfitzmann R [8]	2007	Retrospective Cohort	Europe	51.2	290	56	23	89
Vieira A [76]	2007	Retrospective Cohort	Other(Brazil)	47	17	2	-	29.6
Wells JT [77]	2007	Retrospective Cohort	Europe	50.3	148	24	20	90.5
Gedaly R [23]	2008	Retrospective Cohort	US-Canada	52	142	27	-	41.2
Immordino G [17]	2009	Retrospective Cohort	US-Canada	53.2	110	13	-	-
Tandon P [26]	2009	Retrospective Cohort	US-Canada	52	171	41	22	64.8
Biselli M [78]	2010	Retrospective Cohort	Europe	48	49	13	3	58
Chen GH [79]	2010	Retrospective Cohort	Asia Pacific	52.8	16	1	-	32.4
DiMartini A [80]	2010	Prospective Cohort	US-Canada	52	208	95	-	-
Karim Z [81]	2010	Retrospective Cohort	US-Canada	50.5	80	8	8	-
Hartl J [82]	2011	Retrospective Cohort	Europe	52.5	109	17	-	31
Mathurin P [7]	2011	Prospective Cohort	Europe	47.4	26	3	2	20
Schmeding M [9]	2011	Retrospective Cohort	Europe	48.9	271	73	73	-
Staufer K [83]	2011	Prospective Cohort	Europe	53.5	141	28	-	-
Faure S [84]	2012	Retrospective Cohort	Europe	51	206	90	50	81.7
Addolorato G [85]	2013	Retrospective Cohort	Europe	49.4	92	22	-	-
Deruytter E [86]	2013	Retrospective Cohort	Europe	56	108	31	17	55
Kawaguchi Y [87]	2013	Retrospective Cohort	Asia Pacific	52	13	1	-	38
Park YH [19]	2013	Retrospective Cohort	Asia Pacific	52	18	3	2	57
Rice JP [88]	2013	Retrospective Cohort	US-Canada	49.3	300	48	16	82
Rodrigue JR [107]	2013	Retrospective Cohort	US-Canada	55	118	40	12	55
Egawa H [16]	2014	Retrospective Cohort	Asia Pacific	35	140	32	21	44
Grąt M [90]	2014	Retrospective Cohort	Europe	46	66	22	-	88.8
Piano S [91]	2014	Prospective Cohort	Europe	60	23	5	-	-
Dumortier J [92]	2015	Retrospective Cohort	Europe	47.2	712	128	128	63
Hasanin M [93]	2015	Cross-sectional	US-Canada	-	45	8	-	-
Satapathy SK [94]	2015	Retrospective Cohort	US-Canada	54	148	16	16	112.8
Zhou M [28]	2015	Retrospective Cohort	US-Canada	54.2	35	6	-	-
Askgaard G [95]	2016	Retrospective Cohort	Europe	54	156	35	35	-
Hajifathalian K [96]	2016	Prospective Cohort	Europe	56	19	4	-	40.8
Im GY [97]	2016	Retrospective Cohort	US-Canada	41	9	2	1	24.5
Kollmann D [98]	2016	Retrospective Cohort	Europe	-	382	16	-	73
Lee BP [99]	2017	Retrospective Cohort	US-Canada	51.4	31	11	7	19.2
Onishi Y [100]	2017	Retrospective Cohort	Asia Pacific	46	7	1	-	60
Wigg AJ [101]	2017	Retrospective Cohort	Asia Pacific	50	87	18	14	52

### The incidence of alcohol relapse

The characteristics of the studies and the data on alcohol relapse rates are detailed in Table 3. A total of 86 cohort studies with 8061 patients reported incidences of alcohol relapse at any time after LT. The mean age of patients ranged from 35 to 60 years, and the mean follow-up time was 10 to 112 months. The alcohol relapse rate varied across studies with a range of 4 to 95% with an I^2^ of 90.7%. A random effect model was applied and yielded the pooled alcohol relapse rate of 22% (95% CI: 19–25%) during the mean follow-up time of 48.4 ± 24.7 months. The rate of heavy alcohol relapse varied markedly across studies with an I^2^ of 85% and pooled rate of 14% (95%CI: 12–16%).

### Pooled risk factors of alcohol relapse

The effects of all of the risk factors on alcohol relapse after LT that were classified by demographic, risk behavior, social, and comorbidity factors; these were pooled in 37 cohort studies (Table 4). The results of pooling these effects are summarized in Table 5. The results showed that psychiatric comorbidities, pre-transplant abstinence less than 6 months, being unmarried, and smoking were significantly associated with alcohol relapse after LT with corresponding pooled ORs of 3.46 (95% CI: 1.87–6.39), 2.76 (95%CI: 2.10–3.61), 1.84 (95%CI: 1.39–2.43), and 1.72 (95%CI: 1.21–2.46), respectively. In addition, the I^2^ ranged from 0 to 40.6%, with the highest I^2^ in psychiatric comorbidities.

**Table 4:** Summary of the included studies reported risk factors in the meta-analysis

Author	Year	Demographic factors	Risk behavior factors	Social factors	Comorbidity
Kumar S [31]	1990	-	-	Abstinence < 6months	-
Osorio RW [37]	1994	Male Unmarried Unemployed	Substance use	Abstinence < 6months Rehabilitation	Psychiatric disease
Gerhardt TC [38]	1996	-	-	Abstinence < 6months	-
Tringali RA [39]	1996	-	-	Abstinence < 6months	-
Foster PF [24]	1997	Family history of alcohol use	Substance use	Abstinence < 6months Rehabilitation	-
Lucey MR [45]	1997	Male	-	Abstinence < 6months	-
Shakil AO [47]	1997	Male	-	-	-
Tang H [52]	1998	Male	-	-	-
Conjeevaram HS [53]	1999	Male	-	-	-
Newton SE [55]	1999	-	Substance use	-	-
Pageaux GP [18]	1999	Male Unmarried Unemployed	-	Abstinence < 6months	-
Burra P [59]	2000	Unmarried Family history of alcohol use	Substance use Alcohol dependence	Rehabilitation	-
Jain A [61]	2000	-	-	Abstinence < 6months Rehabilitation	-
Mackie J [15]	2001	Male Unmarried Lack of social support Low SES Family history of alcohol use	Smoking	Abstinence < 6months	-
Jauhar S [14]	2004	Male Unmarried Unemployed	Substance use	Abstinence < 6months Rehabilitation	Psychiatric disease
Björnsson E [20]	2005	-	-	Rehabilitation	-
Cuadrado A [10]	2005	Male	-		-
Hwang S [71]	2006	-	-	Abstinence < 6months	-
Kelly M [72]	2006	Unmarried Lack of social support Unemployed	Substance use	Abstinence < 6months	Psychiatric disease Depression
De Gottardi A [29]	2007	Age < 50 years Male Unmarried Low SES Unemployed	High HRAR	Abstinence < 6months	Psychiatric disease
Nickels M [75]	2007	Age < 50 years Male	Alcohol dependence	-	Depression
Pfitzmann R [8]	2007	Age < 50 years Male Unmarried	-	Abstinence < 6months	-
Karim Z [81]	2010	Age < 50 years Male Unmarried Low SES Unemployed	Smoking Substance use	Abstinence < 6months Rehabilitation	Psychiatric disease
Hartl J [82]	2011	-	Smoking	Abstinence < 6months Rehabilitation	-
Addolorato G [85]	2013	-	-	Rehabilitation	-
Deruytter E [86]	2013	Age < 50 years Male Unmarried Unemployed Family history of alcohol use	Smoking Alcohol dependence	-	Psychiatric disease
Kawaguchi Y [87]	2013	Male	High HRAR	-	-
Park YH [19]	2013	Male	-	Abstinence < 6months	-
Rice JP [88]	2013	Male	-	-	-
Rodrigue JR [89]	2013	Lack of social support	Smoking	Abstinence < 6months Rehabilitation	-
Egawa H [16]	2014	Male Unmarried Lack of social support Unemployed	Smoking High HRAR	Abstinence < 6months	Psychiatric disease
Zhou M [28]	2015	-	High HRAR	-	-
Askgaard G [95]	2016	Male Unmarried Unemployed Family history of alcohol use	Smoking Alcohol dependence	Abstinence < 6 months	Depression
Im GY [97]	2016	Male Unmarried Family history of alcohol use	Smoking	-	Psychiatric disease
Lee BP [99]	2017	Male	-	-	-
Onishi Y [100]	2017	Age < 50 years Male	-	-	-
Wigg AJ [101]	2017	Male Unmarried Lack of social support Unemployed Family history of alcohol use	Smoking Substance use	Rehabilitation	Psychiatric disease

**Table 5:** Pooled risk factors of alcohol relapse

Factors	N	OR	95%CI	Pooling method	I^2^	Egger test (P-value)
Demographic factors						
Age < 50 years	6	1.16	0.43 - 3.15	Random effect	75.2	0.55
Sex (male)	23	0.89	0.69 - 1.11	Fixed effect	21.7	0.43
Unmarried	14	1.84	1.39 - 2.43	Fixed effect	14.6	0.57
Lack of social support	5	1.78	0.72 - 4.38	Random effect	49.5	0.18
Low SES	3	0.99	0.15 - 6.50	Random effect	86.3	0.28
Unemployed	10	1.33	0.93 - 1.89	Fixed effect	7.7	0.74
Family history of alcohol use	7	1.49	0.94 - 2.36	Fixed effect	23.0	0.50
Risk behavior factors						
Smoking	9	1.72	1.21 - 2.46	Fixed effect	0	0.69
Substance use	8	1.06	0.48 - 2.34	Random effect	58.5	0.71
Alcohol dependence	4	1.22	0.43 - 3.40	Random effect	61.8	0.15
High HRAR	4	2.93	0.30 - 28.64	Random effect	79.6	0.18
Social factors						
Abstinence < 6 months	20	2.76	2.10 - 3.61	Fixed effect	18.1	0.02
Rehabilitation program	11	1.10	0.59 - 2.04	Random effect	67	0.71
Comorbidity						
Psychiatric disease	9	3.46	1.87 - 6.39	Random effect	40.6	0.02
Depression	3	2.13	0.49 - 9.25	Random effect	54.4	0.60

*N* Number, *OR* Odds ratio, *CI* Confidence interval, *I*^*2*^ I^2^statistics, *SES* Socioeconomic status, *HRAR* High-risk alcohol relapse scale

### Subgroup analysis

**Fig. 2:**
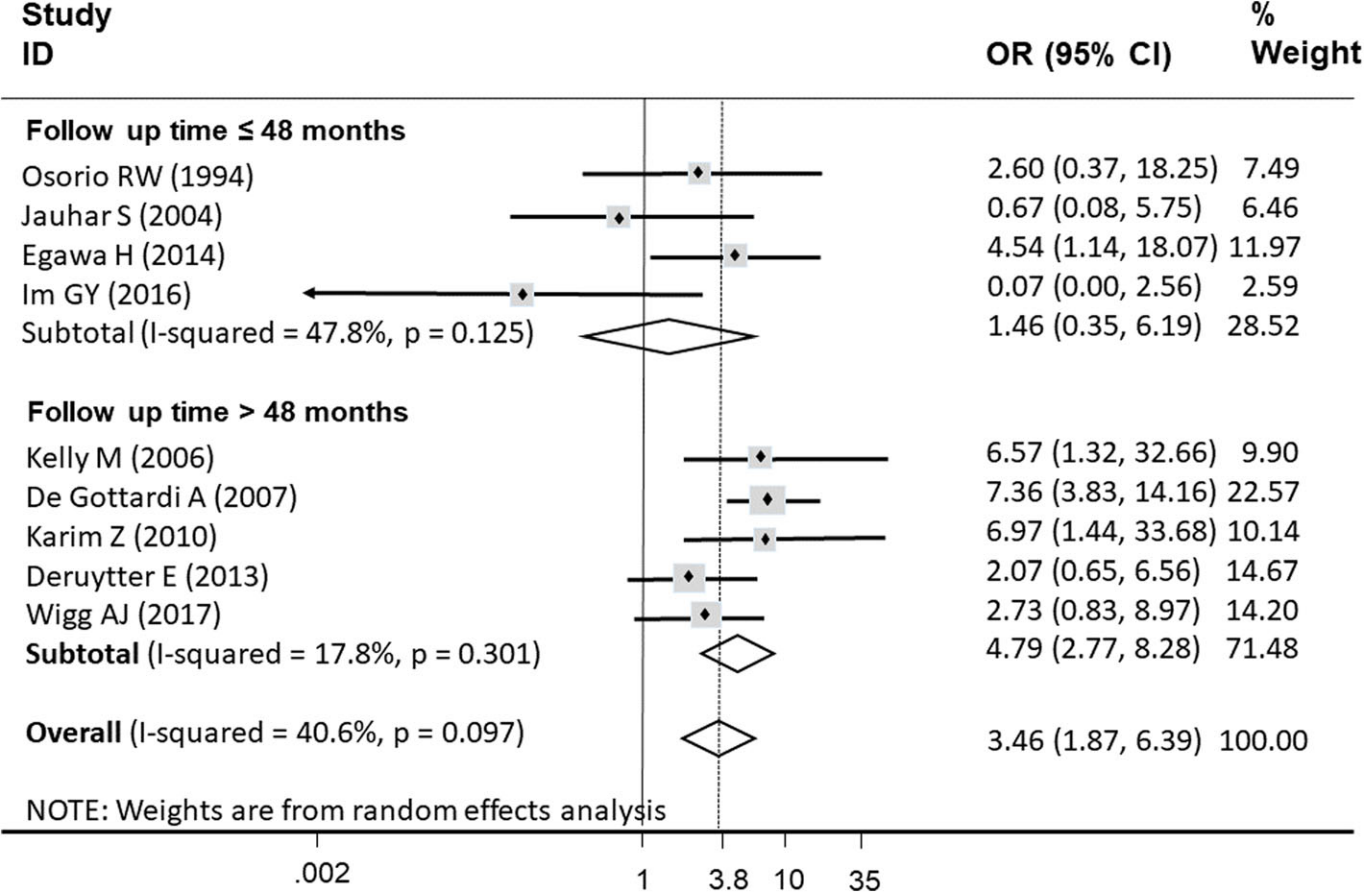
Subgroup analysis of duration of follow-up time in psychiatric comorbidity factor

Subgroup analysis by age (≤ 50 years or > 50 years), regions of studies (Europe, North America, Asia Pacific and Brazil), definition of alcohol relapse (only report or report combining with biochemical testing), and follow-up time (≤ 4 years or > 4 years) was performed to explore the potential cause of heterogeneity of pooled rates of alcohol relapse and heavy alcohol relapse. Likewise, the subgroup analysis was performed with psychiatric comorbidities to identify the factor associated with alcohol relapse with the highest risk and heterogeneity. Subgroup analyses showed no significant difference in all analyses of alcohol relapse and heavy alcohol relapse rates except for one analysis of psychiatric comorbidities. Patients with psychiatric comorbidities who had longer follow-up time (> 4 years) had an increased risk of alcohol relapse versus those with a shorter follow-up time (≤ 4 years) (Fig. 2).

### Publication bias

**Fig. 3:**
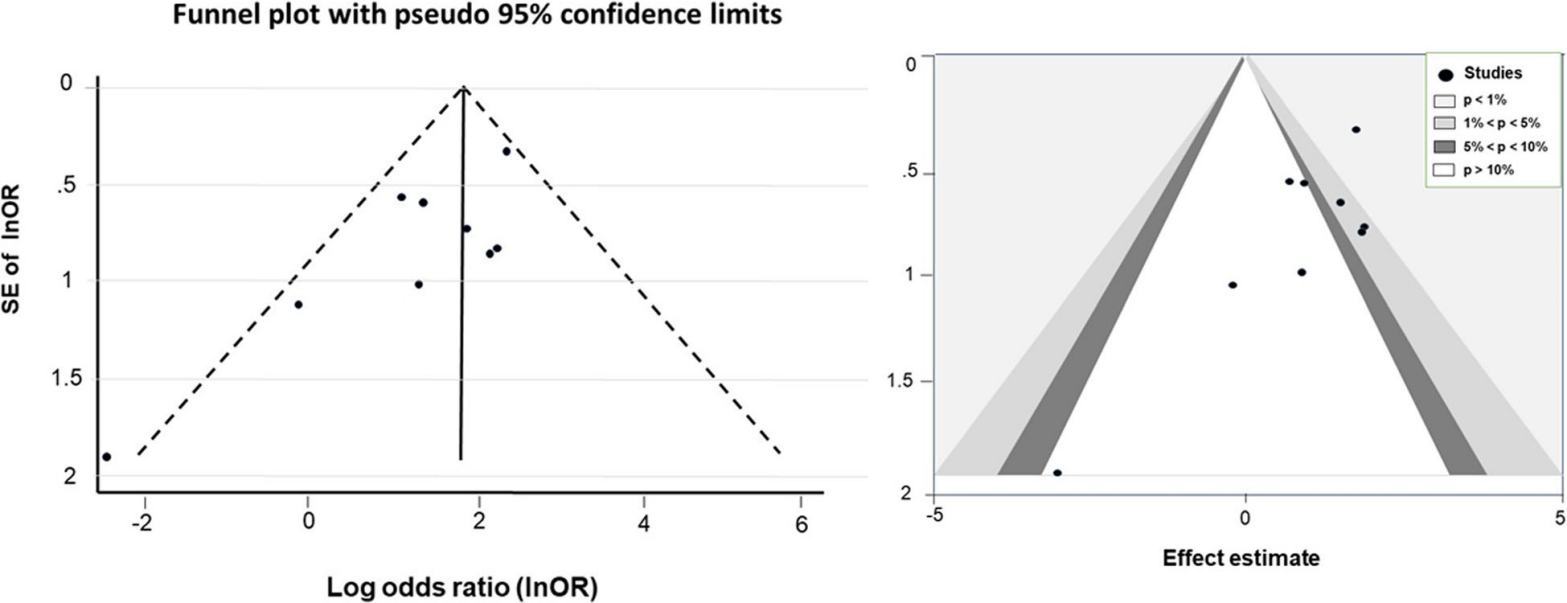
Funnel plot and contour-enhanced funnel plot for psychiatric comorbidities

**Fig. 4:**
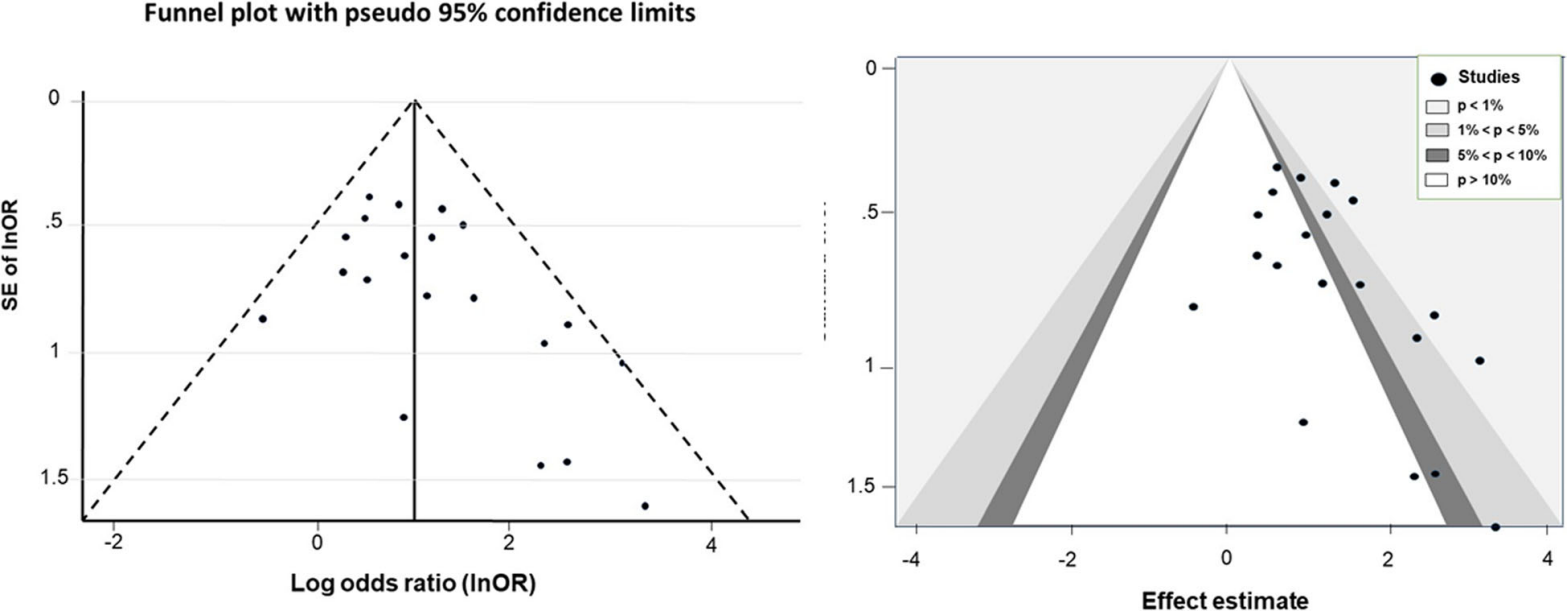
Funnel plot and contour-enhanced funnel plot for less than 6 months of pre-transplant abstinence

The Egger test showed no evidence of publication bias among the studies, and the shape of the funnel plots was symmetrical in all analyses except for psychiatric comorbidities (Fig. 3) and abstinence less than 6 months (Fig. 4). The studies that reported less than 6 months of abstinence were both non-significant and significant leading to a contour-enhanced funnel plot; thus, asymmetry may not be due to either publication bias or heterogeneity. The studies with negative effect of psychiatric co-morbidities and abstinence less than 6 months were not reported.

## Discussion

Alcohol relapse after LT remains an ethical issue in LT for alcoholic liver disease due to its harmful and negative impacts on liver grafts. One meta-analysis and four systemic reviews of alcoholic liver disease in LT patient were published earlier [25, 103–106]. The well-designed meta-analysis of rate and risk factors of alcohol relapse by Dew et al. in 2008 included 54 studies published between 1983 and 2005 [25]. A systematic review by Rustard et al. in 2015 selected only articles of the risk factors of alcohol relapse [103]. A systematic review by McCallum et al. in 2006 included only studies that were associated with psychosocial criteria [106]. Bravata et al. performed a systematic review of alcohol relapse and evaluated only the association between employment aspect and alcohol relapse [105]. One systematic review focused on neither alcohol relapse rate nor risk factor [104]. Our study is a systematic review and meta-analysis of all published studies up to 2018, which aimed to estimate post-LT alcohol relapse rate and its predictive factors. To date, the current study is the most extensive meta-analysis of alcohol relapse in LT patients.

Our study demonstrated that any alcohol relapse and heavy alcohol relapse rates were as high as 22 and 14% during the mean follow-up time of 48.4 ± 24.7 months, respectively. The literature on alcohol relapse post-transplantation has reported a wide range of alcohol relapse rates, which might be due to different definitions of alcohol relapse. Dew et al. reported that the average rate for alcohol relapse after LT was 5.6 cases per 100 patients per year for any alcohol relapse and 2.5 cases per 100 patients per year for heavy alcohol relapse [25]. The authors suggested that a significant proportion of patients who returned to any alcohol drinking then became heavy drinkers, which led to a significant harm to LT recipients [25].

In our study, the most significant risk factors of relapse were psychiatric comorbidities followed by pre-transplant alcohol abstinence less than 6 months, being unmarried, and smoking. Four of the nine studies reported that psychiatric conditions had a link with alcohol relapse [16, 29, 72, 81]. The finding was consistent with the previous meta-analysis [25]. The study identified 3 of the 12 psychosocial variables associated with any alcohol relapse: < 6 months abstinence prior to transplant, poor social support, and a family history of alcohol abuse or dependence [25]. We found that 9 of the 20 studies revealed that alcohol abstinence less than 6 months was associated with alcohol relapse [8, 18, 29, 37, 81, 82, 95, 107, 108]. Our study confirms the validity of using the 6-month rule of alcohol abstinence as a criterion for pre-transplant selection in patients with ALD; this is consistent with the previous meta-analysis study [25].

A systematic review of large prospective studies focusing on risk factors for alcohol relapse following LT has also suggested that a shorter length of pre-transplant sobriety was a significant predictor of alcohol relapse [103]. However, the 6-month rule cannot be applied in LT for patients with severe acute alcoholic hepatitis whose condition is not allowed to wait until 6 months. LT in this group of patients remains a controversial issue in many transplant centers. The current data do not suggest that LT in patients with severe alcoholic hepatitis leads to more alcohol relapse [109]. Therefore, 6 months of alcohol abstinence may not reliably predict post-LT alcohol relapse. Other risk factors were psychiatric comorbidities, a high score on the HRAR scale, and a diagnosis of alcohol dependence [103]. Scoring systems to predict alcohol relapse after LT such as HRAR and the ARRA were proposed for use, but they have never been validated by well-designed studies.

In this study, psychiatric co-morbidities and pre-transplant abstinence less than 6 months were strong predictive factors of alcohol relapse with some publication bias against negative studies. Psychiatric comorbidities were the strongest risk factor in this study but with high heterogeneity. Interestingly, subsequent subgroup analysis showed that longer follow-up times led to an increased impact of psychiatric comorbidities on any alcohol relapse. The psychiatric comorbidities defined in enrolled studies included all psychiatric conditions that could cause impaired daily functioning, i.e. anxiety, schizophrenia, and personality disorders. In this study, we analyzed three cohort studies that reported depression separately because depression is a known risk factor associated with alcoholic drinking. We found that depression was not a significant factor in alcohol relapse (OR = 3; 95%CI 0.49–9.25).

Clinical practice has changed considerably since the first studies that recruited in 1988. The differences in the definition of alcohol relapse and heavy relapse as well as a lack of objective means of documenting alcohol use in these studies are limitations. Furthermore, heavy alcohol relapse was defined only in some studies (Table 2). Including unpublished studies may solve this problem. The absence of negative studies of psychiatric co-morbidities and abstinence less than 6 months likely caused publication bias. However, this attempt cannot guarantee a reasonably low heterogeneity after including unpublished studies.

## Conclusions

We demonstrated the pooled rates of any alcohol relapse and heavy alcohol relapse post-LT. Furthermore, we identified predictive factors of alcohol relapse after LT to be used during the selection process of LT candidates. With respect to the prevention of alcohol relapse post-LT, alcohol abstinence of at least 6 months, appropriate screening and care of psychiatric co-morbidities, and smoking cessation should be incorporated in pre-transplant selection and management periods. Careful selection of LT candidates and modifying pre-transplant risk factors of alcohol relapse has the potential to reduce alcohol relapse after LT.

## Data Availability

Not applicable.

## Abbreviations

ALD: Alcoholic liver disease
CI: Confidence interval
HRAR: High-Risk Alcohol Relapse
LT: Liver transplantation
OR: Odds ratio
